# Endobronchial endometriosis presenting as central-type lung cancer: a case report

**DOI:** 10.1186/1746-1596-8-53

**Published:** 2013-04-03

**Authors:** Juan-Han Yu, Xu-Yong Lin, Liang Wang, Yang Liu, Chui-Feng Fan, Yong Zhang, En-Hua Wang

**Affiliations:** 1 Department of Pathology, the First Affiliated Hospital and College of Basic Medical Sciences, China Medical University, Shenyang 110001, China; 2 Institute of pathology and pathophysiology, China Medical University, Shenyang 110001, China

**Keywords:** Endometriosis, Bronchus, Pathology, Immunohistochemistry

## Abstract

**Abstract:**

A 45-year-old female patient was referred to our hospital for complaining of dyspnea and coughing in the past four months. The computed tomography scanning demonstrated a central lesion in the upper lobe of the left lung close to the hilar, and the subsequent bronchoscopy revealed a polypoid lesion of the distal of the left main bronchus. This patient was diagnosed clinically as “possibly central-type lung cancer”. However, the pathologic result of the surgically excised polypoid lesion was endobronchial endometriosis.

**Virtual Slides:**

The virtual slide(s) for this article can be found here: http://www.diagnosticpathology.diagnomx.eu/vs/1077439085928525

## Background

Endometriosis is defined as the presence of endometrial tissue, including endometrial glands and stroma, in the body areas out of the uterus. Endometriosis foci are usually located in the pelvis and abdomen, and rarely in the thorax. Moreover, the thoracic endometriosis commonly affects the lung parenchyma, pleura, and diaphragm, and the prominent clinical manifestations are recurrent hemoptysis, pneumothorax, hemothorax, and asymptomatic pulmonary nodules [1,2]. We herein report a rare case of endobronchial endometriosis presenting as central-type lung cancer.

## Case presentation

**Figure 1:**
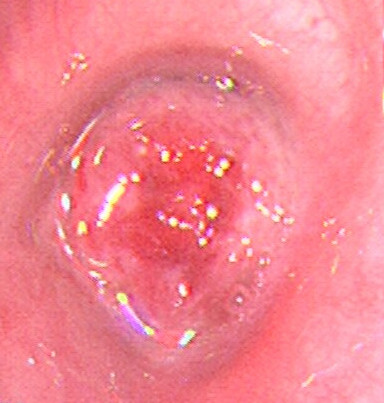
Bronchoscopic examination showing a pink-red polypoid lesion blocked the left main bronchus.

A 45-year-old female without a history of smoking was referred to our hospital for complaining of dyspnea and coughing in the past four months. The patient had no fever or haemoptysis. No prior history of dysmenorrhea, dyspareunia, or pelvic pain was found as well. The physical examination showed the left lung breath sounds weakened compared with contralateral. On computed tomography (CT) scanning, a round-like lesion of 1.5 × 1.7 cm in size was observed in the upper lobe of the left lung close to the hilar, and the diagnosis was “possible of central-type lung cancer”. The bronchoscopy (BF-1 T260, Olympus, Tokyo, Japan) revealed a polypoid lesion in the distal of the left main bronchus, and the lumen was blocked (Figure 1). The bronchoscopic diagnosis was “possible of central-type lung cancer” as well. Pathologic result of bronchoscopic biopsy showed that there was only some squamous epithelium. Thus, the patient underwent the lobectomy in our hospital. No adjuvant treatment was performed and the patient was found well without recurrence at 2 years after surgery.

## Materials and methods

The resected specimens were fixed with 10% neutral-buffered formalin and embedded in paraffin blocks. Tissue blocks were cut into 4-μm slides, deparaffinized in xylene, rehydrated with graded alcohols, and immunostained with the following antibodies: cytokeratin (CK, AE1/AE3), thyroid transcription factor 1 (TTF-1, 8G7G3/1), ER (SP1), CD10 (56C6), and Ki67 (MIB-1) (MaiXin, China). Sections were then stained with a streptavidin-peroxidase system (KIT-9720, Ultrasensitive TM S-P, MaiXin, China). The chromogen used was diaminobenzidine tetrahydrochloride substrate (DAB kit, MaiXin, China). All the samples were slightly counterstained with hematoxylin, dehydrated, and mounted. For the negative controls, each sample was incubated with PBS instead of the primary antibody as above described.

## Results

**Figure 2:**
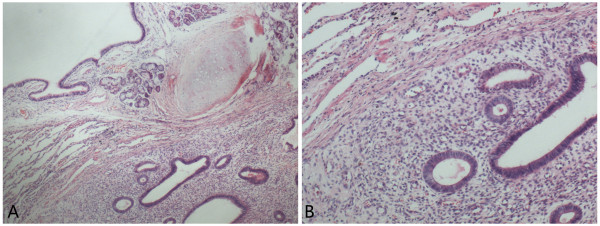
**The endometrial-like glands and stromal cells were found under the bronchial epithelium (A).** The ectopic glandular structures surrounded by a densely distributed endometrial-like stroma, and the glands predominantly showed a single layer of columnar cells similar to the endometrial epithelial lining with basal nuclei, and no marked cytological atypia could be observed (**B**). A, HE×100; B, HE×200.

Grossly, the resected lung tissue was about 14.0×4.5×3.0 cm in size. The polypoid lesion (1.5×1.3×1.0 cm in size) was located in the bronchus and the cut surface showed a pink-red color. Histologically, the normal bronchial epithelium, submucosal glands, cartilage, and alveolar epithelium were found in the section. Of note, there were some ectopic glandular structures surrounded by densely distributed endometrial-like stromal cells under the bronchial mucosa. The ectopic glands predominantly showed a single layer of columnar cells similar to the endometrial epithelium lined with the basal nuclei, and no marked cytological atypia was observed. The mitosis was rare (Figure 2).

**Figure 3:**
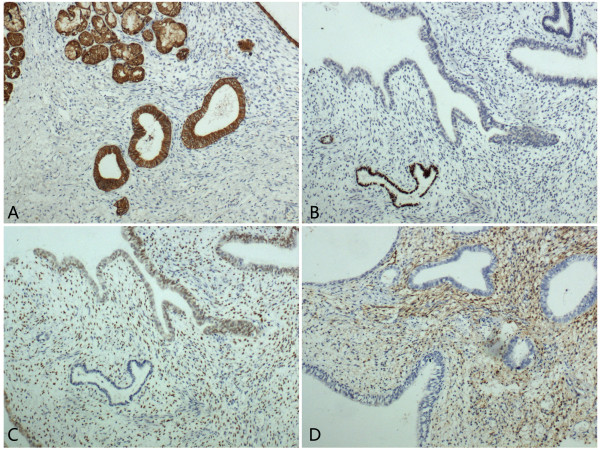
**The bronchial epithelium and the glands were positive for CK, while the stromal cells were negative (A).** The alveolar epithelial cells were positive for TTF-1 (**B**). The ectopic glands and the endometrial-like stromal cells were positive for ER (**C**). The endometrial-like stromal cells were positive for CD10 (**D**). A-D, SP×200.

Immunohistochemical staining showed that all the epithelium and the glands were positive for CK, while the remaining alveolar epithelial cells were positive for TTF-1. The ectopic glands showed positive staining for ER, and the densely distributed stromal cells were positive for both ER and CD10 (Figure 3). Ki67 index was less than 5%. According to the morphological and immunohistochemical findings, the final diagnosis was endobronchial endometriosis.

## Discussion

The occurrence of endometriosis in areas other than the abdomen and pelvis is uncommon, even rare in the thorax. Thoracic endometriosis is usually located in the lung parenchyma, pleura, and diaphragm. The endometriosis of the bronchus is extremely rare. To our knowledge, there were only five cases of thoracic endometriosis reported to occur in the bronchus previously [2-6]. The typical clinical manifestations of thoracic endometriosis include pneumothorax (72%), hemoptysis (14%), or hemothorax (12%) coincident with menstrual bleeding, and only 2% cases were presented as pulmonary nodules [7,8]. Moreover, catamenial hemoptysis was documented in all the five patients with endobronchial endometriosis [2-6], suggesting that patients with tracheal endometriosis are prone to hemoptysis. However, in our case, the patient only showed dyspnea and coughing without hemoptysis, and the case history gave no other valuable information.

The CT scanning and bronchoscopy, which have been considered to be valuable in diagnosing thorax endometriosis, both have a yield in this extremely rare condition. It was reported that asymptomatic pulmonary nodules was documented in only 2% patients with thoracic endometriosis [7]. While of the 5 cases with tracheal endometriosis, no patient presented as pulmonary nodules [2-6]. In our case, CT scanning detected a round-like lesion in the upper lobe of the left lung close to the hilar. The bronchoscopy revealed that there was a polypoid lesion in the distal of the left main bronchus. Accordingly, the clinical diagnosis was considered as “possibly central-type lung cancer”. Though the bronchoscopic biopsy was performed, the result was of little value in precluding neoplasm of the lung. Therefore, surgical treatment was imperative.

On pathology sections, endometrial glands and stromal cells were found under the bronchial epithelium. Furthermore, the immunohistochemistry was performed to confirm the diagnosis. The immunohistochemical results revealed that all the epithelium and the glands were positive for CK, while the stromal cells were negative. All the epithelium and glandular structures which were positive for CK showed no atypia. The alveolar epithelial cells were positive for TTF-1. The ectopic glands showed positive staining for ER, and the stromal cells were positive for both ER and CD10. Taken together, the possibility of pulmonary tumors exhibiting atypical microscopic changes could be ruled out [9,10], and the diagnosis of this case is endobronchial endometriosis.

In this case, the patient underwent lobectomy, which could not prevent recurrence. Cancer antigen CA-125 may be useful to monitor the progress of endometriosis [11,12].

## Conclusion

Reports of endobronchial endometriosis are extremely rare. Diagnosis of etiology remains challenging due to the absence of specific clinical characteristics, especially when the features of CT scanning and bronchoscopy could not exclude the possibility of central-type lung cancer. In this condition, to make a right diagnosis can only depend on pathological examination. Endobronchial endometriosis had to be considered in the differential diagnosis of “central-type lung cancer”, particularly in patients with known endometriosis.

## Consent

Written informed consent was obtained from the patient for publication of this case report and accompanying images. A copy of the written consent is available for review by the Editor-in Chief of this Journal.

## Competing interests

The authors declare that they have no competing interests.

## Authors’ contributions

YJH and LXY participated in the histopathological evaluation, performed the literature review, acquired photomicrographs and drafted the manuscript. FCF and LY carried out the immunohistochemical stains evaluation. ZY conceived and designed the study. WEH gave the final histopathological diagnosis and revised the manuscript. WL edited the manuscript. All the authors read and approved the final manuscript.
